# i-Rheo-Indent: Broadband rheological characterisation of soft solids by macroscale indentation

**DOI:** 10.1007/s00397-026-01590-7

**Published:** 2026-08-05

**Authors:** Andrea Jannina Fernandez, Simeon Skopalik, Claire Smart, Graham M. Gibson, María del Pilar Noriega Escobar, Manlio Tassieri

**Affiliations:** 1 Division of Biomedical Engineering, James Watt School of Engineering, Advanced Research Centre, University of Glasgow, Glasgow, G11 6EW UK; 2 Soapworks Ltd, Coltness St., Glasgow, G33 4JD UK; 3 School of Physics and Astronomy, Advanced Research Centre, University of Glasgow, Glasgow, G11 6EW UK; 4 Daabon Group, Santa Marta, Colombia

**Keywords:** Rheology, Indentation, Linear viscoelasticity, Soft solids, Broadband rheology, Fourier transform, Mechanical characterisation

## Abstract

**Abstract:**

We introduce *i-Rheo-Indent*, a transformation-based methodology for determining the linear viscoelastic properties of soft solids directly from macroscale indentation experiments. By combining force-relaxation and indentation-depth measurements with direct time-to-frequency transformations, the method recovers the complex shear modulus, $G^{*}(\omega )$, over a broad frequency range without requiring oscillatory excitation or predefined constitutive models. The methodology addresses key limitations of conventional rotational rheometry, where compressional forces required to maintain sample–tool contact may alter the measured response through stress stiffening and interfacial artefacts. Stress-relaxation indentation experiments were performed using a rigid truncated-conical indenter, with the geometry explicitly incorporated into the constitutive formulation. The approach was validated across hydrogels, polydimethylsiloxane elastomers, anatomical modelling materials, and industrial soap bar formulations. Good agreement with oscillatory rheometry was obtained over a broad frequency range, while improved consistency with capillary rheometry was observed for highly structured soft solids under Cox–Merz comparison. These results establish *i-Rheo-Indent* as a simple, rapid, and robust methodology for broadband rheological characterisation of soft solids.

**Graphical Abstract:**

**Figure:**
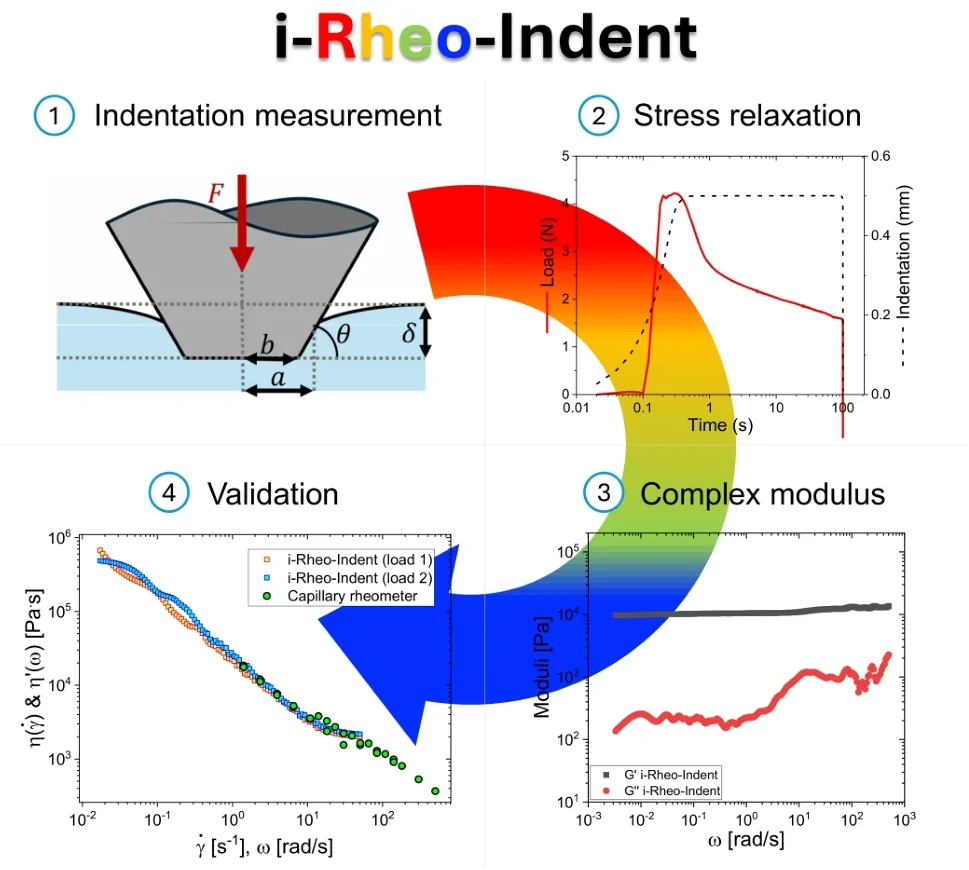

## Introduction

The quantitative determination of the linear viscoelastic properties of soft solids remains one of the major experimental challenges in rheology. While conventional rotational rheometers have long represented the standard instrumentation for measuring the viscoelastic response of complex materials, these systems were originally designed and optimised primarily for fluids and soft viscoelastic liquids rather than for highly structured soft solids (MacSporran and Spiers 1982; Costanzo et al. 2024). Consequently, when applied to materials such as hydrogels (Meyvis et al. 1999), elastomers (Barrès et al. 2003; Walter et al. 2017), biological tissues (Nasseri et al. 2002; Holt et al. 2008; Cirka et al. 2012), anatomical surrogates (Parkins et al. 2021; Holt et al. 2011), soaps (Castro et al. 2010; Joshi et al. 2026), and synthetic detergent formulations (Ciccone et al. 2022), conventional rheometric approaches often encounter substantial experimental and conceptual limitations.

A first major challenge concerns sample preparation and loading. Unlike liquids, soft solids cannot easily conform to the geometry of the rheometer fixtures, making it difficult to guarantee reliable and reproducible contact between the sample and the measuring tools. To overcome this issue, a compressional normal force is commonly applied during sample loading by reducing the rheometer gap until sufficient adhesion and contact stability are achieved. However, as recently demonstrated in our studies, this seemingly unavoidable procedure may itself significantly perturb the mechanical state of the material and alter the measured viscoelastic response. In particular, soft hydrogels and biomimetic extracellular-matrix analogues were shown to exhibit pronounced compressional stress stiffening or softening when subjected to normal stresses perpendicular to the imposed shear deformation (Ciccone et al. 2020; Ferraro et al. 2023). These effects become particularly problematic when relatively small rheometric geometries are employed, as is often the case for biological samples or expensive biomaterials where the available sample volume is limited. Under these conditions, the compressional stress applied during sample loading may easily drive the material outside its linear viscoelastic regime, thereby compromising the reliability of the measurements (Ferraro et al. 2023). Moreover, additional artefacts such as wall slip (Walter et al. 2017; Walls et al. 2003), edge fracture (Tanner and Keentok 1983; Keentok and Xue 1999; Inn et al. 2005), sample debonding (Archer et al. 1997), and geometric confinement (Cullen et al. 2003) may further affect the rheological characterisation of soft solids, especially for highly structured or heterogeneous materials.

The importance of overcoming these limitations has grown substantially over the past decade due to the increasing recognition that the viscoelastic properties of soft biological materials play a fundamental role in mechanobiology, tissue engineering, regenerative medicine, and cancer research (Chaudhuri et al. 2015; Grolman et al. 2020; Adebowale et al. 2021). Indeed, the mechanical properties of biological tissues and tissue-mimicking materials are now widely recognised as key regulators of cellular behaviour, including migration, adhesion, proliferation, differentiation, and mechanotransduction pathways (Mierke 2021; Dewig et al. 2022; Banche-Niclot et al. n.d.).

In particular, recent studies have highlighted that not only the elastic properties, but also the viscous and dissipative components of soft biological matrices strongly influence cell fate and tissue functionality. For example, it has been shown that stem-cell chondrogenesis can be regulated through the viscous component of isoelastic hydrogels, demonstrating that cells actively respond to the time-dependent mechanical relaxation of their surrounding microenvironment (Chaudhuri et al. 2016; Walker et al. 2024). Similarly, the viscoelastic properties of multicellular spheroids and tumour tissues are increasingly recognised as potential biomechanical fingerprints capable of distinguishing healthy tissues from pathological counterparts and of providing insight into tumour progression, metastasis, and therapeutic resistance (Ferraro et al. 2024b; Courbot and Elosegui-Artola 2025; Banche-Niclot et al. n.d.).

Despite this growing interest, the rheological characterisation of soft tissue surrogates and biological specimens remains experimentally difficult. Traditional techniques such as rotational rheometry, atomic force microscopy (AFM) (Walker et al. 2024), nanoindentation (Akhtar et al. 2018), microfluidics (Del Giudice 2022), optical tweezers (Tassieri 2019), and compression assays (Ferraro et al. 2024a) often require sophisticated instrumentation, complex geometrical assumptions, or highly specialised expertise. Furthermore, many of these techniques remain strongly dependent on the imposed geometry and boundary conditions, making direct comparison between methods difficult (Ciccone et al. 2022).

Over the past decade, alternative approaches have emerged that seek to extract rheological properties directly from time-domain measurements, bypassing many of the limitations associated with conventional rheometric geometries. Among these, the *i-Rheo* framework (Tassieri et al. 2016) introduced a transformation-based methodology capable of recovering broadband viscoelastic spectra directly from experimentally measured signals without relying on predefined constitutive models. Initially developed for step-strain rheology, the *i-Rheo* philosophy was subsequently extended to microrheology (Tassieri 2019), AFM indentation (Chim et al. 2018), optical assays (Ferraro et al. 2024b), and more recently to inverse transformations enabling the reconstruction of time-domain relaxation functions from frequency-domain data (Ramírez and Tassieri 2026).

A key conceptual insight underlying these developments is that linear rheology can be formulated as a transformation problem: the intrinsic material response is encoded in the relationship between conjugate variables, such as force and deformation, and can therefore be recovered independently of the specific experimental protocol provided that the appropriate transformation framework is employed.

In this work, we extend this philosophy to macroscale indentation and introduce *i-Rheo-Indent*, a methodology that combines controlled indentation experiments with direct transformation techniques to recover the linear viscoelastic properties of soft solids over a broad frequency range. Unlike conventional rheometry, where compressional forces required for sample loading may inadvertently alter the material response, the present approach incorporates the compressive deformation directly into the constitutive framework itself. The methodology is implemented using a rigid truncated-conical indenter mounted onto a conventional mechanical testing platform operating in compression mode, as described in the associated patent application (Tassieri and Skopalik 2026).

The proposed methodology enables broadband rheological characterisation from a single stress-relaxation experiment, without requiring oscillatory excitation, predefined viscoelastic models, or conventional rheometric geometries. Moreover, because the deformation is imposed locally through indentation rather than through boundary-driven shear, the method is intrinsically less sensitive to wall slip, imperfect sample–tool contact, and compressional stress artefacts.

We demonstrate the validity and robustness of the *i-Rheo-Indent* methodology across a broad range of materials, including hydrogels, silicone elastomers, anatomical model materials, and industrial soft solids such as soap bars formulations. In particular, for the soap and syndet (from **syn**thetic **det** ergent) systems investigated during the development of the associated patent, *i-Rheo-Indent* was found to yield remarkably improved agreement with capillary rheometry measurements under Cox–Merz comparison relative to conventional rotational rheometry. These findings strongly support the hypothesis that many of the discrepancies previously observed in conventional rheometry originate predominantly from geometry- and contact-related artefacts.

Ultimately, *i-Rheo-Indent* establishes a new transformation-based framework for the rheological characterisation of soft solids, extending the broader *i-Rheo* philosophy beyond conventional rheometric geometries and providing a robust methodology for probing the viscoelastic properties of increasingly important biomimetic and biological materials.

## Materials and Methods

### Materials

The *i-Rheo-Indent* methodology was validated on a broad range of soft solids spanning different material classes, including physically crosslinked hydrogels, silicone elastomers, commercially available anatomical model materials, and industrial cleansing bars. This diverse selection of samples was specifically chosen to assess the robustness and general applicability of the methodology across systems exhibiting markedly different viscoelastic properties, microstructures, and processing histories.

Soft hydrogel samples were prepared using gelatin and agar at different concentrations in order to generate materials with tunable viscoelastic properties spanning more than one order of magnitude in storage modulus. Gelatin gels were fabricated at concentrations of 6, 10, and 15 wt.% by first heating deionised water to $50\,^{\circ }\textrm{C}$. Gelatin powder (Sigma Aldrich G2500) was then weighed and dissolved into the warm water under continuous magnetic stirring. The mixtures were stirred for an additional 30 minutes at $50\,^{\circ }\textrm{C}$ to ensure complete dissolution before being poured into moulds, producing samples with thicknesses of at least 5 mm for indentation measurements. The solutions were subsequently left at room temperature until complete gelation had occurred (Skopinska-Wisniewska et al. 2021; Serafin et al. 2023).

Agar gels were prepared at concentrations of 2, 3, and 4 wt.% by dispersing weighed agar powder (Sigma Aldrich 01916) into deionised water. The mixtures were magnetically stirred for approximately 2 hours at $100\,^{\circ }\textrm{C}$ to ensure complete dissolution of the agar before being cast into moulds. The resulting samples were then allowed to cool and solidify at room temperature prior to testing (Zhang et al. 2025).

Polydimethylsiloxane (PDMS) elastomers were prepared using Dow SILGARD^TM^ 184 silicone elastomer kits. The elastomer base and curing agent were mixed at ratios of 5:1, 10:1, and 20:1 (base-to-curing-agent ratio) for approximately 10 minutes, thus enabling the generation of samples with progressively increasing compliance. The mixtures were subsequently poured into moulds, degassed under vacuum for 30 minutes to remove trapped air bubbles, and cured in an oven at $60\,^{\circ }\textrm{C}$ for 1 hour prior to testing.

For all hydrogel and PDMS samples, additional specimens suitable for oscillatory shear rheometry were prepared by casting the materials into moulds designed to produce disk-like samples with thicknesses of approximately 1.5 mm.

In addition to laboratory-prepared soft solids, commercially available materials designed for anatomical modelling and biomedical simulation (Stratasys Ltd.) were also investigated. These included materials based on different blends of TissueMatrix^®^, Agilus30^™^+TissueMatrix^®^, radiology phantom formulations based on TissueMatrix^®^ and VeroClear^™^, and synthetic subcutaneous fat analogues based on GelMatrix^™^. The materials were fabricated by multi-material 3D printing using a Stratasys Digital Anatomy^™^ printer into approximately cubic specimens of dimensions $2 \times 2 \times 2~\mathrm {cm^{3}}$, with the compositions and internal architectures preset through the printer software. Following stress-relaxation indentation measurements, the cubes were sectioned into cylindrical specimens having thicknesses of approximately 1.5 mm for oscillatory shear rheometry measurements. These materials were selected because they represent highly relevant examples of soft solids whose mechanical properties are commonly characterised using indentation-based techniques in biomedical and surgical simulation applications.

A further class of materials investigated in this work consisted of solid cleansing bars based on synthetic detergent formulations (syndets). These materials were selected because they represent an important industrial example of highly structured soft solids for which conventional rheological characterisation is particularly challenging, especially under processing conditions relevant to extrusion and moulding operations (Ciccone et al. 2022). In particular, three representative commercial samples were investigated in the present study. The first consisted of the syndet base SB5 (Syndopal 300MB, Stephenson Group Ltd., Leeds, UK), which served as the reference base material. In addition, two commercially available syndet-based formulations were investigated: *Kiss me Argan Shampoo Bar* (KMA) and *Seas The Day* (STD). Owing to commercial confidentiality agreements, the detailed formulations of the latter two materials cannot be disclosed. For rotational rheometry and *i-Rheo-Indent* measurements, the materials were thermally conditioned at approximately $40\,^{\circ }\textrm{C}$ and subsequently moulded or extruded into geometries compatible with the corresponding experimental setups, following procedures similar to those previously described for syndet formulations (Ciccone et al. 2022).

### Rotational rheometry

**Fig. 1:**
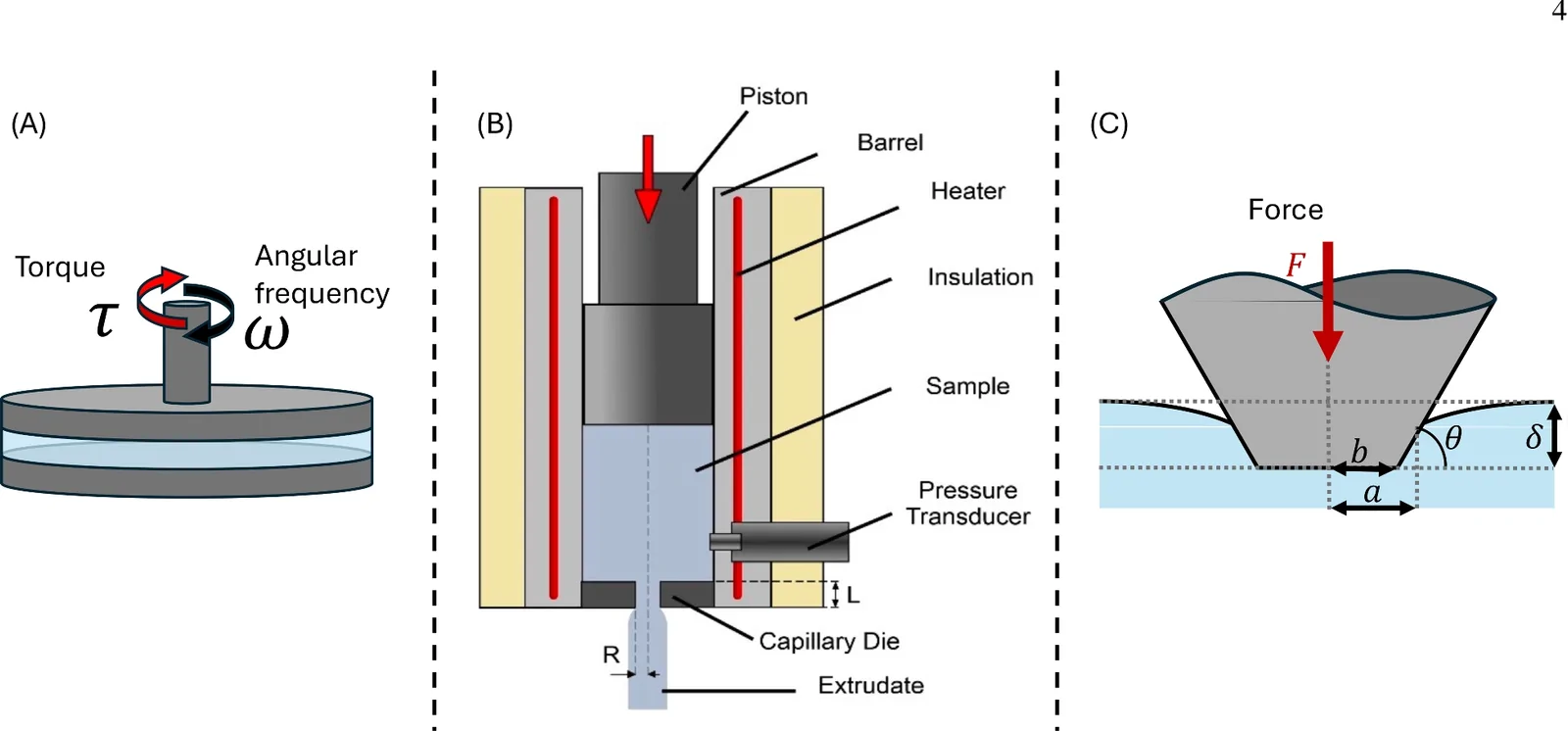
Schematic comparison of the rheological methodologies employed in the present work. (**A**) Conventional rotational rheometry, where oscillatory shear deformation is imposed between parallel plates through an applied torque *τ * at angular frequency *ω *. (**B**) Twin-bore capillary rheometry, in which the material is extruded through a capillary die by means of a piston-driven barrel system equipped with pressure transducers and thermal control, enabling determination of the steady shear viscosity under processing-relevant flow conditions. (**C**) Schematic representation of the *i-Rheo-Indent* methodology introduced in this work, where a rigid truncated-conical indenter is driven into the sample while the force relaxation response *F*(*t*) and indentation depth *δ (t)* are continuously recorded. The geometric parameters of the indenter, including the effective contact radius *a*, blunt-end radius *b*, and cone angle *θ *, are explicitly incorporated into the constitutive formulation used to recover the complex viscoelastic spectrum from the time-domain indentation experiment

Conventional oscillatory shear rheology measurements were performed in order to provide benchmark linear viscoelastic properties for comparison with the *i-Rheo-Indent* methodology. Measurements were carried out using a stress-controlled rheometer (Anton Paar Physica MCR 302 Instruments) equipped with interchangeable parallel-plate geometries, schematically illustrated in Fig. 1(A), having diameters of 8 mm, 15 mm and 25 mm, referred to as PP08, PP15 and PP25, respectively. For most measurements, a 15 mm parallel-plate geometry was employed (Ferraro et al. 2023). Unless otherwise stated, all rheological measurements were performed at room temperature ($20\pm 2\,^{\circ }\textrm{C}$). For soap bars and syndet formulations, measurements were instead carried out under controlled thermal conditions at approximately $40\,^{\circ }\textrm{C}$ in order to reproduce the industrial processing and extrusion conditions.

The linear viscoelastic properties of the investigated materials were characterised in terms of the complex shear modulus:

1 $$\begin{aligned} G^*(\omega )=G'(\omega )+iG''(\omega ) \end{aligned}$$

where *G'(ω )* and *G''(ω )* are the storage and loss moduli, respectively.

Frequency sweep measurements were performed within the materials’ linear viscoelastic regime over a discrete range of angular frequencies spanning approximately three decades, from 0.1 to $100~\mathrm {rad\,s^{-1}}$. Prior to frequency sweep measurements, amplitude sweep tests were performed in order to identify the linear viscoelastic regime of each material.

However, as previously demonstrated for soft hydrogels and structured soft solids, the application of compressional stresses perpendicular to the imposed shear deformation can itself significantly alter the measured rheological response (Ciccone et al. 2020, 2022; Ferraro et al. 2023). In particular, our earlier investigations on syndets and soap bars revealed substantial stress-induced stiffening effects, with both the storage modulus and apparent yield stress increasing by up to two orders of magnitude as the applied normal stress increased (Ciccone et al. 2022). Moreover, increasing discrepancies were observed between the dynamic viscosities measured by oscillatory rheometry and the shear viscosities obtained via capillary rheometry under conditions where larger compressive stresses were required to maintain sample–tool contact.

These observations strongly suggest that, in highly structured soft solids, conventional rotational rheometry may perturb the material state itself through the very compressional forces required to perform the measurement. This limitation provided one of the principal motivations for the development of the *i-Rheo-Indent* methodology, where the imposed compressive deformation becomes an intrinsic and controlled component of the rheological measurement rather than an unavoidable experimental artefact.

### Capillary rheometry

Capillary rheometry measurements were performed on selected soap and syndet formulations in order to characterise their nonlinear shear-flow behaviour under conditions more representative of industrial extrusion processes.

Measurements were carried out using a twin-bore capillary rheometer (Rosand 2000, Netzsch, Germany), schematically illustrated in Fig. 1(B), following the procedures described in Ref. (Ciccone et al. 2022). Samples were extruded under isothermal conditions at $40\,^{\circ }\textrm{C}$ through capillary dies of different diameters and aspect ratios depending on the formulation under investigation.

The apparent wall shear stress and apparent shear rate were calculated according to standard capillary rheometry relations:

2 $$\begin{aligned} \sigma _{\textrm{app}}= \frac{(P_0-P_L)R}{2L} \end{aligned}$$

3 $$\begin{aligned} \dot{\gamma }_{\textrm{app}}= \frac{4Q}{\pi R^3} \end{aligned}$$

where $(P_0-P_L)$ is the pressure drop along the capillary, *R* and *L* are the capillary radius and length, respectively, and *Q* is the volumetric flow rate. Rabinowitsch corrections were subsequently applied in order to account for the non-Newtonian character of the materials (Wilczyński 2020).

The capillary rheometry measurements served as an important reference for comparing the rheological response obtained under steady shear flow with that measured under oscillatory deformation. In particular, the Cox–Merz empirical rule was adopted to directly compare the complex viscosities obtained from oscillatory shear rheometry with the steady shear viscosities measured by capillary rheometry for the same soap and syndet formulations. However, as shown in Fig. 8 of our previous work on synthetic detergent formulations (Ciccone et al. 2022), satisfactory agreement between the two methods could not be achieved. Significant discrepancies were consistently observed between the dynamic and steady shear viscosities, with the disagreement becoming increasingly pronounced under conditions where larger compressive loads were required to maintain reliable contact between the sample and the rheometer fixtures during oscillatory measurements.

These observations represented one of the principal motivations for the development of the *i-Rheo-Indent* methodology, where the compressive deformation is no longer treated as an unavoidable experimental artefact, but rather becomes an intrinsic and controlled component of the rheological measurement itself. As will be shown in the Results section, when the same Cox–Merz comparison was performed using *i-Rheo-Indent* measurements, remarkably improved agreement with capillary rheometry was obtained.

### i-Rheo-Indent measurements

The *i-Rheo-Indent* methodology was implemented using a macroscale indentation system specifically designed to acquire force-relaxation data during controlled compressive indentation of soft solids.

The experimental setup consisted of a universal testing machine (either a Mecmesin MultiTest 2.5 or OmniTest 2.5) equipped with interchangeable rigid indenters and low-capacity load cells of either $2~\textrm{N}$ or $250~\textrm{N}$, selected according to the stiffness and force response of the investigated material. A schematic representation of the indentation geometry and experimental principle is shown in Fig. 1(C). The materials were placed onto a rigid platform while a rigid indenter was driven vertically into the sample under displacement-controlled conditions.

Measurements were performed using a truncated-conical indenter geometry, as introduced in the associated patent application (Tassieri and Skopalik 2026). The use of a truncated cone was found to provide robust and reproducible measurements across a broad range of soft solids, while simultaneously reducing uncertainties associated with contact-area determination and sample confinement. The experimental platform could additionally incorporate a temperature-controlled sample holder, allowing rheological measurements to be performed under controlled thermal conditions. This aspect was particularly important for the syndet formulations investigated in this work, whose viscoelastic response strongly depends on temperature and processing conditions relevant to industrial extrusion and moulding operations (Tassieri and Skopalik 2026). Consistent with the rotational rheometry measurements, indentation experiments were performed under the same temperature conditions described above.

During each experiment, a rigid truncated-conical indenter having a base diameter of $8~\textrm{mm}$, a blunt-end diameter of $0.5~\textrm{mm}$, and an inclination angle of $60^{\circ }$ was brought into gentle contact with the sample surface until a small preload force exceeding approximately $0.01~\textrm{N}$ was detected, thereby ensuring reproducible initial contact conditions. A finite compressional step strain was then applied by imposing indentation depths typically ranging between $10~\mathrm {\mu m}$ and $500~\mathrm {\mu m}$, depending on the stiffness and characteristic relaxation behaviour of the investigated material. Following the imposed indentation, the subsequent force-relaxation response *F*(*t*) and the corresponding indentation depth *δ (t)* were continuously recorded with high temporal resolution. The duration of each experiment was selected according to the relaxation dynamics of the sample, such that the force response could be monitored over a sufficiently long time window to approach a quasi-equilibrium condition.

One practical advantage of the proposed methodology is that the sample geometry is considerably less restrictive than in conventional rheometry. Unlike parallel-plate measurements, the sample does not need to conform to a precisely defined geometry or completely fill the gap between two measuring fixtures. Instead, reliable measurements can be obtained provided that the specimen dimensions are sufficiently larger than both the imposed indentation depth and the characteristic dimensions of the indenter, thereby minimising boundary effects. In the present work, all samples satisfied these conditions. Care was nevertheless taken to ensure intimate contact between the specimen and the supporting substrate, avoiding air gaps that could affect the measured response. Overall, sample loading is substantially simpler than in either rotational or capillary rheometry, requiring only that the specimen be positioned beneath the indenter. This simplicity also makes the methodology attractive for future implementation in manufacturing environments, where indentation measurements could potentially be performed directly on materials in production or storage, provided that adequate support and adhesion to the underlying substrate are ensured.

The experimentally acquired force and indentation-depth signals were subsequently analysed within the *i-Rheo* framework, as described in the following section, in order to recover the frequency-dependent viscoelastic properties of the investigated materials directly from the time-domain relaxation response.

## Theoretical framework

The theoretical framework underlying *i-Rheo-Indent* builds on the transformation-based methodology introduced in i-Rheo (Tassieri et al. 2016) and later extended to atomic force microscopy (AFM) indentation experiments within the *i-Rheo-AFM* framework (Chim et al. 2018). Here, this approach is further generalised to macroscale indentation.

Early developments in stress-relaxation measurements under indentation can be traced to Tripathy and Berger (2009), who formulated a viscoelastic description of AFM relaxation experiments by combining the Boltzmann superposition principle with Hertzian contact mechanics. Their analysis, however, relied on fitting the experimental response with prescribed viscoelastic models and associated relaxation spectra. In contrast, *i-Rheo-Indent* employs direct time-to-frequency transformations to extract broadband viscoelastic spectra directly from the measured signals, thereby avoiding any *a priori* constitutive assumptions.

Accordingly, the starting point is the Boltzmann superposition principle for a linear viscoelastic material subjected to an arbitrary indentation history. Following Tripathy and Berger (2009), the indentation force can be expressed as:

4 $$\begin{aligned} F(t)=\int _{-\infty }^{t} G(t-\tau )\,\frac{d\Lambda (\delta (\tau ))}{d\tau }\,d\tau \end{aligned}$$

where *G*(*t*) is the shear relaxation modulus of the material and $\Lambda (\delta (t))$ is the generalized deformation function associated with the indentation depth *δ (t)*.

In the original formulation of Tripathy and Berger (2009), $\Lambda (\delta (t))$ was derived from Hertzian contact mechanics for a rigid spherical indenter acting on an incompressible linear elastic half-space. Under these assumptions, the *instantaneous* deformation function is given by:

5 $$\begin{aligned} \Lambda (\delta (t))=\frac{8}{3}\sqrt{R}\,\delta ^{3/2}(t) \end{aligned}$$

where *R* is the radius of the spherical indenter.

**Fig. 2:**
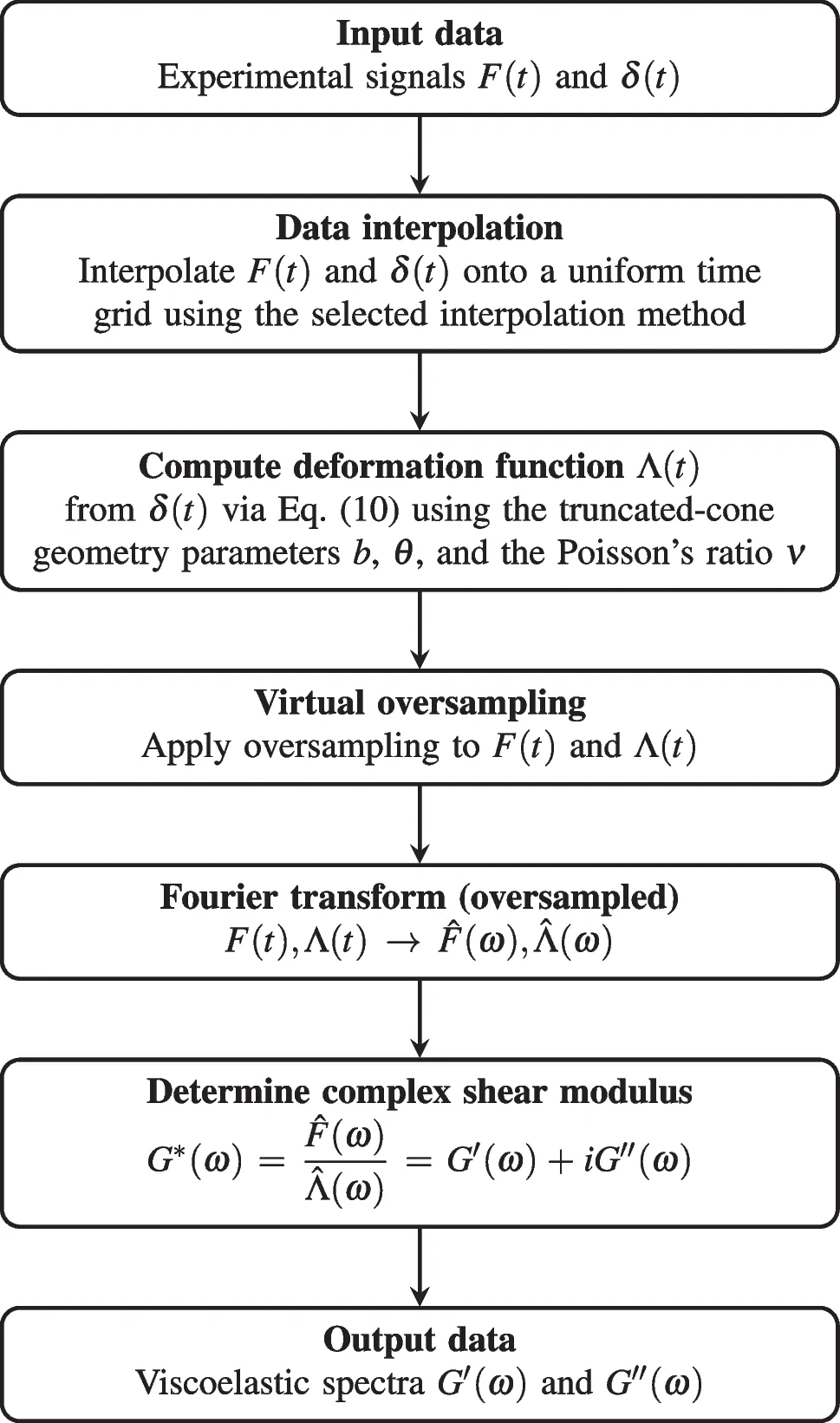
Workflow of the *i-Rheo-Indent* methodology. The experimentally measured force relaxation *F*(*t*) and indentation depth *δ (t)* are first interpolated and subsequently used to calculate the deformation function *Λ (t)* associated with the truncated conical indenter geometry. The resulting signals are virtually oversampled and transformed into the frequency domain, enabling direct determination of the complex shear modulus $G^*(\omega )$ without requiring oscillatory excitation or predefined viscoelastic constitutive models

The present work extends this concept from microscopic AFM indentation to macroscale rheological indentation using a rigid indenter attached to a conventional mechanical testing platform operating in compression mode. In contrast to *i-Rheo-AFM*, where spherical probes were adopted to satisfy classical Hertzian assumptions and minimise cellular penetration effects, the present methodology employs a rigid indenter having the shape of a truncated cone, as described in the associated patent application (Tassieri and Skopalik 2026). This geometry was found to provide significantly improved robustness and reproducibility for soft solids and industrial formulations, particularly in the case of soap bars and structured materials. The overall computational workflow of the *i-Rheo-Indent* methodology, from the acquisition of the experimental force and indentation signals to the determination of the complex shear modulus, is schematically illustrated in Fig. 2.

For a truncated conical indenter, the deformation function *Λ (d)* is no longer described by the classical Hertzian solution derived for spherical indenters. Instead, the geometric contribution to the deformation must explicitly account for the finite blunt radius and inclination angle of the truncated cone. The indentation depth *δ (t)* for a rigid truncated conical indenter acting on an incompressible linearly elastic half-space can be related to the effective contact radius *a*(*t*) through:

6 $$\begin{aligned} \delta (a,b,\theta ,t)=\phi _{0}(t)\,a(t)\,\tan (\theta ), \end{aligned}$$

and

7 $$\begin{aligned} \phi _{0}(t)=\cos ^{-1}\left( \frac{b}{a(t)}\right) \end{aligned}$$

where *b* is the radius of the blunt end of the indenter, *θ * is the inclination angle of the cone.

Under these assumptions, the corresponding *instantaneous* force–indentation relationship becomes:

8 $$\begin{aligned} F(t)= \left( \frac{2G(t)}{1-\nu } \right) \tan (\theta )\, a^{2}(t) \left[ \phi _{0}(t) + \cos (\phi _{0}(t))\sin (\phi _{0}(t)) \right] \end{aligned}$$

where *G*(*t*) is the shear modulus and *ν * is the Poisson’s ratio of the material. From Eq. 8, the material instantaneous deformation function can therefore be identified as:

9 $$\begin{aligned} {\begin{matrix} \Lambda (a,b,\theta ,t)=\left( \frac{2}{1-\nu } \right) \tan (\theta )\, a^{2}(t) \left[ \phi _{0}(t)\right. \\ \left. +\cos (\phi _{0}(t))\sin (\phi _{0}(t))\right] \end{matrix}} \end{aligned}$$

Since the direct experimental determination of the contact radius *a* is generally impractical during indentation experiments, a first-order approximation is introduced by assuming that the material wets the truncated cone up to a height equal to the indentation depth *δ (t)*. Under this approximation, the deformation function becomes explicitly dependent on the experimentally measured indentation depth:

10 $$\begin{aligned} {\begin{matrix} \Lambda (\delta (t),b,\theta )=& \frac{\tan (\theta )}{1-\nu } \left( \delta (t)\,\tan (\theta )+b \right) ^{2}\\ & \times \left\{ \cos ^{-1} \left( \frac{b}{\delta (t)\,\tan (\theta )+b} \right) \right. \\ & \left. +\sin \left[ \cos ^{-1} \left( \frac{b}{\delta (t)\,\tan (\theta )+b} \right) \right] \right. \\ & \left. \times \left( \frac{b}{\delta (t)\,\tan (\theta )+b} \right) \right\} \end{matrix}} \end{aligned}$$

Thus, the experimentally measured indentation depth can be transformed into an effective deformation variable analogous to strain in conventional rheometry, while explicitly incorporating the geometry of the indenter into the constitutive formulation.

Substitution of Eq. 10 into Eq. 4 yields the generalized constitutive equation governing the indentation response of the viscoelastic material, which is formally identical to the constitutive relation previously employed in i-Rheo for shear deformations and in *i-Rheo-AFM* for spherical indentation. Since Eq. 4 is a convolution integral, transformation into the frequency domain directly yields:

11 $$\begin{aligned} \hat{F}(\omega )=G^*(\omega )\,\hat{\Lambda }(\omega ) \end{aligned}$$

and therefore:

12 $$\begin{aligned} G^*(\omega )=\frac{\hat{F}(\omega )}{\hat{\Lambda }(\omega )} \end{aligned}$$

where $\hat{F}(\omega )$ and $\hat{\Lambda }(\omega )$ are the Fourier transforms of the force and deformation functions, respectively.

A key aspect of this framework is that no oscillatory excitation is required. Instead, the full viscoelastic spectrum is reconstructed from a single time-domain indentation experiment, provided that both force and indentation depth are measured with sufficient temporal resolution. This follows directly from the Boltzmann superposition principle, according to which the constitutive equation is expressed as a convolution between the material relaxation modulus and the imposed deformation history. Consequently, within the framework of linear viscoelasticity, the material response can be analysed equivalently in either the time or frequency domain. This concept is analogous to passive microrheology, where the thermal Brownian motion of tracer particles may be interpreted as a sequence of impulsive excitations that collectively probe a broad frequency spectrum. In the present methodology, broadband excitation is instead generated by a step-indentation experiment. Since a Dirac impulse is simply the time derivative of a Heaviside step function, both excitations contain the full frequency spectrum and therefore enable the recovery of the complex viscoelastic modulus through appropriate Fourier-transform methods. As previously demonstrated within the *i-Rheo* framework (Tassieri et al. 2016; Chim et al. 2018), the experimentally accessible frequency window is fundamentally dictated by the temporal limits of the experiment:

13 $$\begin{aligned} \omega _{\min }=\frac{1}{t_{\max }}, \qquad \omega _{\max }=\frac{1}{t_{\min }} \end{aligned}$$

where $t_{\min }$ is associated with the acquisition sampling interval and $t_{\max }$ with the total duration of the relaxation experiment.

To improve numerical stability and minimise discretisation artefacts, the experimentally acquired signals are processed using interpolation and virtual oversampling procedures prior to Fourier transformation, following the same principles previously introduced in *i-Rheo* framework (Tassieri et al. 2016; Chim et al. 2018). These procedures effectively shift Nyquist-related artefacts outside the experimentally relevant frequency window and allow accurate reconstruction of continuous viscoelastic spectra.

Although the present work focuses on a truncated-conical indenter, the proposed framework is not restricted to this specific geometry. As in classical Hertzian indentation, the indenter geometry enters the constitutive formulation exclusively through the geometry-dependent deformation function *Λ *. Consequently, provided that the appropriate deformation function can be derived, the same transformation framework can be extended to alternative indenter geometries, including spherical, conical, pyramidal, or flat-ended indenters. The truncated-conical geometry was adopted here because it provided the most robust and reproducible results across the classes of soft solids investigated, whereas preliminary investigations using the classical spherical formulation did not achieve comparable agreement with conventional rheological measurements. The identification of optimal geometries for specific classes of materials remains an important direction for future developments of the methodology.

Importantly, the present formulation represents a conceptual departure from conventional geometry-dependent rheology. In classical rheometry, the measured response is intrinsically linked to the imposed shear geometry and to boundary conditions at the sample–tool interface. In contrast, the *i-Rheo-Indent* framework treats the experiment as a generic mapping between conjugate variables — force and deformation — from which intrinsic viscoelastic properties are recovered through direct transformation. Consequently, the rheological information becomes fundamentally decoupled from the specific deformation geometry, provided that the geometry-dependent deformation function $\Lambda (\delta (t))$ is correctly defined.

This point is particularly important for soft solids such as soap bars, gels, elastomers, and structured biomaterials, where conventional rotational rheometry may be strongly affected by wall slip, sample fracture, imperfect contact conditions, and stress stiffening induced by the compressional forces required to maintain contact between rheometer fixtures and the sample. In such systems, indentation naturally provides a controlled local deformation field while being substantially less sensitive to many of the boundary-condition artefacts associated with sustained shear flow in conventional rotational rheometry.

Thus, *i-Rheo-Indent* extends the broader *i-Rheo* philosophy beyond traditional rheometric geometries and establishes a general framework in which rheological properties can be extracted directly from arbitrary deformation protocols through appropriate transformation of experimentally measured signals.

## Results & Discussion

**Fig. 3:**
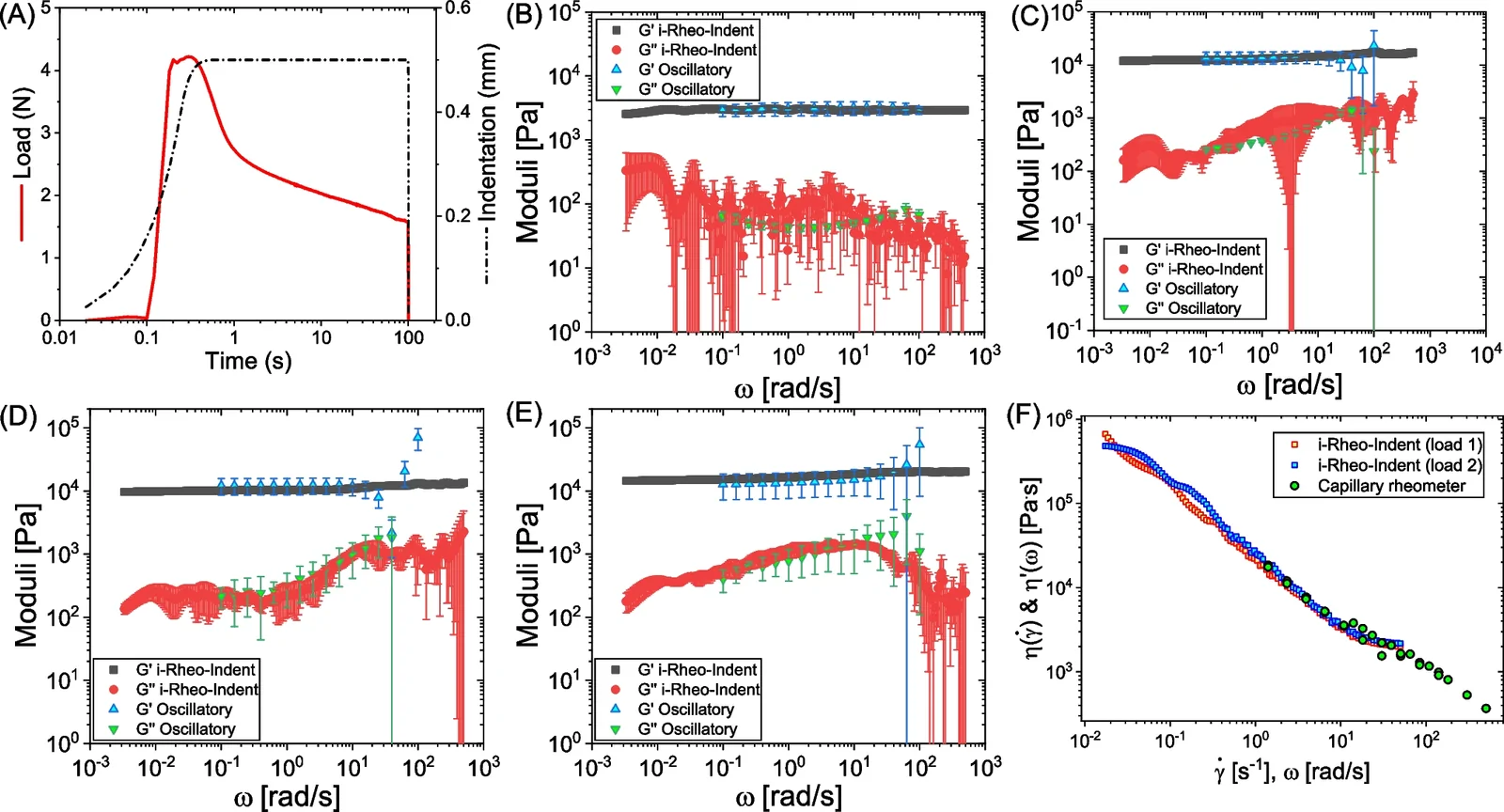
Representative experimental results obtained using the *i-Rheo-Indent* methodology. (**A**) Typical force-relaxation response *F*(*t*) (solid red line) and corresponding indentation depth *δ (t)* (dash-dotted black line) recorded during a step-indentation experiment. Following the rapid imposition of the indentation step, the force exhibits a characteristic viscoelastic relaxation over time while the indentation depth is maintained approximately constant. (B)–(E) Comparison between the viscoelastic moduli obtained by conventional oscillatory rheometry and by *i-Rheo-Indent* for (**B**) a gelatin hydrogel at a concentration of $6~\mathrm {wt.\%}$, (C)–(E) three commercially available materials for anatomical modelling and biomedical simulation, comprising (**C**) a radiology phantom material and (**D**) *Material 1* and (**E**) *Material 4*, two proprietary formulations. In all four cases, quantitative agreement between the two methodologies is observed over the experimentally accessible frequency range. Error bars in panels (B)–(E) represent the standard error calculated over three independent replicates for both measurement techniques. (**F**) Comparison between the steady shear viscosity $\eta (\dot{\gamma })$ measured by capillary rheometry and the dynamic viscosity $\eta '(\omega )$ obtained from *i-Rheo-Indent* measurements for Syndopal 300MB, a structured soft material commonly used in syndet-bar manufacturing. The scatter observed in the capillary rheometry data reflects the experimental variability associated with different loading conditions during extrusion measurements, whereas the *i-Rheo-Indent* data correspond to two independent indentation depths (loadings). The good overlap obtained through the Cox–Merz comparison strongly supports the capability of *i-Rheo-Indent* to recover rheological properties representative of the material response under processing-relevant flow conditions

**Fig. 4:**
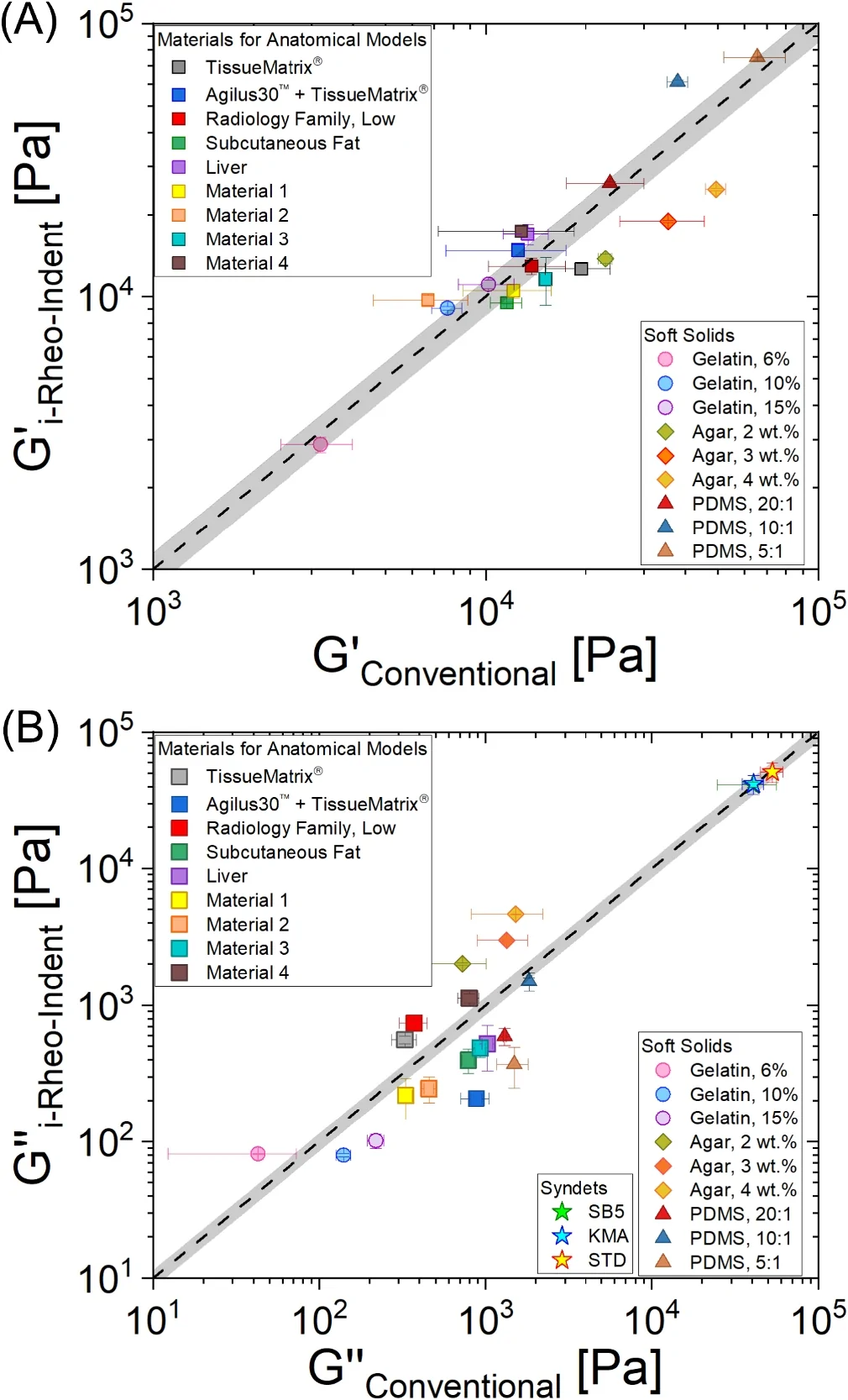
Comparison between the viscoelastic properties measured by conventional rheometry and by *i-Rheo-Indent* across a broad range of soft solids. (**A**) Comparison of the average storage modulus *G'(ω )*, evaluated over the plateau region, obtained by conventional rotational rheometry and by the *i-Rheo-Indent* methodology for hydrogels (gelatin and agar), PDMS elastomers, anatomical modelling and biomedical simulation materials. (**B**) Corresponding comparison of the mean loss modulus *G''(ω )* evaluated over a small range of frequencies spanning from $\omega =0.5~\mathrm {rad\,s^{-1}}$ to $\omega =2~\mathrm {rad\,s^{-1}}$ for the same materials shown in (A) plus the industrial syndet formulations. In both panels, the dashed line represents perfect agreement between the two techniques, while the shaded grey band denotes a deviation of $\pm 15\%$, corresponding to the typical experimental uncertainty associated with rheological measurements. Error bars represent the standard error obtained from three independent measurements for all materials, except for the syndet formulations, for which two independent measurements were performed

The *i-Rheo-Indent* methodology was validated across a broad range of soft solids spanning markedly different compositions, microstructures, and viscoelastic properties, including physically crosslinked hydrogels, silicone elastomers, anatomical modelling materials, and highly structured industrial syndet formulations. Representative experimental results are reported in Fig. 3, while the overall validation of the proposed methodology across all investigated materials is summarised in Fig. 4. For the predominantly elastic soft solids investigated here, the storage modulus *G'(ω )* exhibits only a weak frequency dependence over the experimentally accessible frequency range. Consequently, Fig. 4(A) compares the average values of *G'(ω )*, evaluated over the plateau region, as measured by conventional rotational rheometry and by *i-Rheo-Indent*, providing a representative measure of the elastic response. For comparison, Fig. 4(B) presents the corresponding mean loss modulus *G''(ω )*, evaluated over the frequency interval $0.5 \le \omega \le 2~\mathrm {rad\,s^{-1}}$. Since *G''(ω )* exhibits a stronger frequency dependence than *G'(ω )*, averaging was restricted to this narrow frequency interval centred around $1~\mathrm {rad\,s^{-1}}$.

A typical stress-relaxation indentation experiment is shown in Fig. 3(A). Following the initial contact between the truncated-conical indenter and the sample surface, a finite compressional step indentation was imposed under displacement-controlled conditions. As expected experimentally, the indentation depth *δ (t)* does not correspond to an ideal instantaneous step function, but instead reaches the target indentation over a finite time interval of a few hundred milliseconds. Similarly, a short delay of approximately $10~\textrm{ms}$ was consistently observed in the force acquisition response, primarily associated with the instrumental response time of the load-cell detection system. Nevertheless, these transient effects remained significantly faster than the characteristic relaxation times of the investigated materials, thereby allowing reliable reconstruction of the viscoelastic spectra, as confirmed by the excellent agreement obtained with conventional rheometry.

Depending on the investigated material and on the signal-to-noise ratio of the relaxation response, the total duration of the indentation experiments typically ranged between approximately $100~\textrm{s}$ and $600~\textrm{s}$. According to Eq. 13, these temporal limits directly determine the experimentally accessible frequency window. Consequently, broadband viscoelastic spectra spanning nearly four decades in frequency could be recovered from a single stress-relaxation indentation experiment lasting only a few minutes.

The experimentally measured force relaxation response *F*(*t*) and indentation depth *δ (t)* were subsequently post-processed according to the workflow illustrated in Fig. 2. In particular, the indentation depth was transformed into the geometry-dependent deformation function *Λ (t)* using (10), after which both observables were Fourier transformed and combined through Eq. 12 to recover the complex shear modulus $G^{*}(\omega )$ directly from the time-domain measurements.

Representative comparisons between *i-Rheo-Indent* and conventional oscillatory rheometry are reported in Fig. 3(B)–(E), illustrating the performance of the proposed methodology for a gelatin hydrogel at $6~\mathrm {wt.\%}$ and three commercially available materials for anatomical modelling and biomedical simulation, including a radiology phantom material and two proprietary formulations. In all four cases, good quantitative agreement is observed between the two methodologies over the experimentally accessible frequency range, despite the fundamentally different experimental protocols employed. The consistency of these results across materials spanning a broad range of stiffness further demonstrates the robustness and general applicability of the proposed approach.

The comparatively larger fluctuations observed in the loss modulus *G''(ω )* in Fig. 3(B)–(E) deserve further discussion. Similar behaviour has previously been reported in other applications of the broader *i-Rheo* framework (Chim et al. 2018; Mendonca et al. 2025) and originates from the propagation of experimental noise through the analytical Fourier-transform procedure (Smith et al. 2021). Importantly, in the absence of experimental noise, the *i-Rheo* transformation methodology yields smooth and fully analytical viscoelastic spectra. Thus, the observed fluctuations do not arise from the transformation itself, but rather reflect the finite signal-to-noise ratio of the experimental measurements.

The influence of experimental noise is most pronounced on the less dominant of the two viscoelastic components because, for the same absolute level of noise, its signal-to-noise ratio is intrinsically lower. For the predominantly elastic soft solids investigated here, the storage modulus *G'(ω )* dominates over most of the experimentally accessible frequency range. Consequently, the loss modulus *G''(ω )*, being the smaller contribution, is inherently more susceptible to fluctuations arising from instrumental noise, finite acquisition resolution, and the propagation of experimental uncertainties through the transformation procedure. This behaviour reflects the relative signal-to-noise ratio of the two viscoelastic components rather than an intrinsic limitation of the Fourier-transform methodology. Nevertheless, the agreement between *i-Rheo-Indent* and conventional oscillatory rheometry remains quantitatively very good over the entire experimentally accessible frequency range, with the corresponding values of *G'(ω )* and *G''(ω )* lying within the experimental uncertainties of the two techniques.

Particularly significant results were obtained for the industrial syndet formulations, as shown in Figs. 3(F) and 4(B). As previously discussed, conventional rotational rheometry often fails to provide reliable rheological characterisation for such highly structured soft solids because large compressional forces are required to maintain adequate contact between the sample and the rheometer fixtures. Under these conditions, substantial stress-induced stiffening effects and poor agreement with capillary rheometry are typically observed (Ciccone et al. 2022). In Fig. 3(F), the dynamic viscosity $\eta '(\omega )=G''(\omega )/\omega$ obtained from *i-Rheo-Indent* is compared against the steady shear viscosity $\eta (\dot{\gamma })$ measured using capillary rheometry for Syndopal 300MB formulation through the Cox–Merz relationship. In contrast to previous comparisons obtained using conventional oscillatory rheometry, the agreement between *i-Rheo-Indent* and capillary rheometry is remarkably good across the explored frequency and shear-rate range. The scatter observed in the capillary rheometry measurements primarily reflects the experimental variability associated with repeated loading procedures and extrusion conditions within the capillary barrels.

The robustness and generality of the methodology across all investigated materials are summarised in Fig. 4, where the viscoelastic moduli obtained by *i-Rheo-Indent* are directly compared with those measured by conventional rheometry. Remarkably, the data collapse closely around the ideal one-to-one correspondence line over more than two decades in maginitude and across all investigated material classes. This result strongly supports the general validity of the transformation-based framework underlying *i-Rheo-Indent*, demonstrating that the recovered rheological properties are largely independent of the specific deformation geometry employed during the experiment.

Importantly, the error bars associated with measurements obtained using conventional rotational rheometry were consistently larger than those obtained using *i-Rheo-Indent*, in some cases by approximately one order of magnitude. These observations strongly support the hypothesis that the compressional stresses required to maintain adequate sample–tool contact during conventional rheometry measurements may themselves perturb the material state and consequently alter the measured viscoelastic response.

Like any rheological technique, *i-Rheo-Indent* measures the average mechanical response over the volume interrogated by the indenter. Consequently, the method is expected to provide representative bulk properties provided that the characteristic length scale of any material heterogeneities remains significantly smaller than the characteristic dimensions of the contact area. This condition was satisfied for all materials investigated in the present study, including the intrinsically heterogeneous commercial syndet formulations, as evidenced by the excellent agreement obtained with capillary rheometry. Likewise, the present formulation assumes that the material remains mechanically invariant throughout the duration of the experiment, consistent with the assumptions underlying linear viscoelasticity. Materials undergoing significant structural evolution during the measurement, such as rapidly phase-separating or strongly ageing systems, therefore fall outside the current scope of the methodology and would require experimental protocols specifically adapted to their evolution timescales. Finally, the highest experimentally accessible frequencies are ultimately determined by the combined effects of the instrumental response time and the fastest relaxation processes present in the material. For the systems investigated here, the good agreement with conventional rheometry across the explored frequency range indicates that these instrumental limitations, including possible inertial or elastic ringing effects, do not influence the reported results.

Beyond the quantitative agreement obtained with both rotational and capillary rheometry, one of the principal advantages of *i-Rheo-Indent* lies in its simplicity, speed, and limited sample requirements. Broadband characterisation by conventional oscillatory rheometry requires sequential measurements over a discrete set of frequencies, together with careful sample preparation, loading, gap adjustment, identification of the linear viscoelastic regime, and cleaning of the rheometer fixtures. For the frequency range investigated here, these procedures typically required experimental times approximately 30 times longer than a single *i-Rheo-Indent* measurement. Capillary rheometry remains an important reference technique for characterising materials under processing-relevant shear-flow conditions, but it requires dedicated and relatively expensive instrumentation, experienced operators, several decilitres of material for each loading, and generally multiple loading cycles to construct a complete flow curve. In addition, the loading, thermal equilibration, cleaning, and reloading of the extrusion barrels and capillary dies can dominate the total experimental time, particularly for adhesive or highly structured materials such as syndets and polymer melts, for which cleaning may require several hours. By contrast, *i-Rheo-Indent* requires only a small pill-shaped specimen and a single stress-relaxation experiment lasting a few minutes to recover viscoelastic spectra spanning nearly four decades in frequency. The method therefore provides substantial reductions in both sample consumption and experimental time, while avoiding the sequential frequency sweeps required in oscillatory rheometry and the corrections commonly applied in capillary rheometry, such as the Rabinowitsch correction for non-Newtonian flow. Combined with the excellent agreement obtained with capillary rheometry for the syndet formulations investigated, these practical advantages highlight the potential of *i-Rheo-Indent* as a rapid and efficient approach for the broadband rheological characterisation of highly structured soft solids.

## Conclusions

We have introduced *i-Rheo-Indent*, a transformation-based methodology for the broadband rheological characterisation of soft solids directly from stress-relaxation indentation experiments. By combining controlled macroscale indentation with direct time-to-frequency transformations, the method enables recovery of the complex viscoelastic spectrum without requiring oscillatory excitation, predefined constitutive models, or conventional rheometric geometries.

The methodology was validated across a broad range of materials, including hydrogels, PDMS elastomers, anatomical modelling materials, and industrial syndet formulations. Good agreement was obtained with conventional rotational rheometry, while substantially improved consistency with capillary rheometry was observed for highly structured soft solids, supporting the hypothesis that many discrepancies in conventional rheometry originate from contact-related and compressional-stress artefacts.

Beyond its accuracy, *i-Rheo-Indent* offers significant practical advantages in terms of speed, simplicity, reproducibility, and reduced sample requirements. Broadband viscoelastic spectra spanning nearly four decades in frequency can be obtained from a single experiment lasting only a few minutes, using small pill-shaped samples and without the need for corrections typically required in capillary rheometry.

More broadly, the present work further reinforces the central idea underlying the *i-Rheo* framework: namely, that rheological properties can be recovered through the transformation of conjugate variables largely independently of the specific experimental geometry employed. In this sense, *i-Rheo-Indent* establishes not only a new experimental methodology, but also a general framework for geometry-independent rheological characterisation of soft materials.

Importantly, the simplicity, speed, and robustness of the proposed methodology make it particularly attractive for future biomedical and mechanobiology applications, where reliable mechanical characterisation of soft tissues and tissue-mimicking materials remains experimentally challenging. The ability to rapidly obtain broadband viscoelastic fingerprints from small samples using relatively simple instrumentation opens promising opportunities for the identification of mechanical biomarkers associated with tissue functionality, disease progression, tumour mechanics, and regenerative medicine. In particular, the capability of *i-Rheo-Indent* to probe both the elastic and dissipative components of soft biological materials over broad frequency ranges may provide new opportunities for identifying subtle viscoelastic signatures linked to pathological states and cellular microenvironments.

## Data Availability

The data that support the findings of this study are available from the corresponding author upon reasonable request.
